# Focus on Brain Health to Improve Care, Treatment, and Rehabilitation in Forensic Psychiatry

**DOI:** 10.3389/fpsyt.2019.00840

**Published:** 2019-11-26

**Authors:** Peter Andiné, Henrik Bergman

**Affiliations:** ^1^Centre for Ethics, Law and Mental Health, Department of Psychiatry and Neurochemistry, Institute of Neuroscience and Physiology, Sahlgrenska Academy, University of Gothenburg, Gothenburg, Sweden; ^2^Forensic Psychiatric Clinic, Sahlgrenska University Hospital, Gothenburg, Sweden; ^3^Department of Forensic Psychiatry, National Board of Forensic Medicine, Gothenburg, Sweden; ^4^Unit of Physiotherapy, Department of Health and Rehabilitation, Institute of Neuroscience and Physiology, Sahlgrenska Academy, University of Gothenburg, Gothenburg, Sweden

**Keywords:** forensic psychiatry, brain health, treatment, physical health, cognitive function

## Background

The aim of forensic psychiatric care is to care for, treat and rehabilitate patients back to independent life outside of hospital without recidivism into serious crime. Although the legal regulation of forensic psychiatric care differs from country to country, these patient groups are often distinguished by severe mental illness, a high risk of recidivism, complex rehabilitation and long hospital stays. Within Swedish forensic psychiatry (1) at any one time there are around 1,700 people sentenced to care. These forensic psychiatric patients often have a psychotic disorder, combined with substance use, and are receiving treatment with antipsychotics (2). Treatment often continues for several years (3) and there is a high risk of criminal recidivism after the end of treatment (4). A major problem is that forensic psychiatric care in general lacks evidence for the interventions that are used (5), although clinical guidelines for the treatment of mentally disordered offenders exist (6, 7). In this article we wish to raise the issue of whether forensic psychiatric care can be improved by reflecting on conditions potentially affecting the brain and the concept of brain health in the light of scientific findings concerning brain function, brain plasticity and clinical function in patients. Brain health may be regarded as a condition in which the brain can perform its functions in the best possible way. Recommended interventions for brain health are often associated with lifestyle changes, such as physical exercise (8), but can actually be assumed to apply to all interventions that aim to make it easier for the brain to function properly. We want to encourage forensic health professionals to perform interventions with potential for improving patients’ brain health, and we want to inspire new research studying the effects of such interventions.

Impaired brain health in forensic psychiatric patients may have a number of different causes and result in different effects on the body, the psyche and on behavior. Some of the obvious causal factors that are often seen in patients include early and long-lasting, extensive substance use, and a psychotic disorder with persistent negative or cognitive symptoms. Many patients also have a background of repeated skull trauma, suspected brain damage due to alcohol misuse in the mother during pregnancy, birth injuries and neuropsychiatric problems that first appeared in childhood. In addition to this, there is a lifestyle of smoking, an unhealthy diet and low levels of physical activity, leading to physical illnesses such as diabetes and arteriosclerosis, which can affect cerebral blood flow. Most of these causal factors are general, but some can also be considered specific. How do we assess a possible influence on brain health in forensic psychiatric patients? One obvious way is the presence of any psychiatric diagnosis that includes cognitive effects. Although some forensic psychiatric patients have neurocognitive diagnoses (9), the number of unrecorded cases may be high, particularly for mild and marginal intellectual disability. In Sweden neuropsychological tests are often performed in connection with a forensic psychiatric examination, while neurobiological examinations such as EEG, brain imaging, functional MRI or blood flow measurements are performed more rarely and then primarily if a tumour, bleed or dementia is suspected. We contend that there are great possibilities, based on existing knowledge and using current methodology, for increasing our knowledge of brain health in forensic psychiatric patients and thus introducing targeted measures for improving function, preventing further functional loss, and compensating for already existing functional loss. In the light of both old knowledge and new research, we would like here to provide a few examples of causes of impaired brain health and examples of interventions that can improve brain health in forensic psychiatric patients.

## Psychotic Disorders

Fundamental to improving brain health among forensic psychiatric patients is to treat any psychotic disorder correctly. Forensic psychiatric patients with a psychotic disorder are often at an increased risk of aggressive behavior and treatment resistance to antipsychotics. For psychotic disorders a number of changes in the brain structure have been described, although these changes are small and varied, and are primarily demonstrated at group level. Individuals with schizophrenia and aggressive behavior, compared with individuals with schizophrenia but without aggression, have smaller volumes in several brain regions, such as the prefrontal cortex (10, 11), a region that plays a key role in cognitive impulse control (12). Treatment-resistant schizophrenia, a common clinical challenge in forensic psychiatry, is associated with even greater frontotemporal changes but perhaps also with specific brain changes (13). These brain changes may explain the greater severity of disorders with negative and cognitive symptoms, as seen in psychotic patients who are difficult to treat (14).

## Substance Use

The link between substance use, brain disorder, mental illness and violent crime is indisputable, and a large part of forensic psychiatric rehabilitation is based on offering a drug-free environment and preventing a relapse into substance use. Alcohol misuse produces brain damage (15) and affects cognitive functions (16), while narcotic preparations can produce brain damage in single doses (17). This itself is obvious, but clinically there is seldom more detailed knowledge of any brain damage and cognitive difficulties the patient may have. Although the patients are often deemed to be generally marked by previous severe substance use, a more detailed mapping of damage and functional difficulties using brain imaging and neuropsychological tests might provide guidance in forensic psychiatric rehabilitation.

## Traumatic Brain Injury

The incidence of traumatic brain injuries increases the risk of violent crime (18), and produces an increased risk of violence when forensic psychiatric patients are in institutional care (19). Some of the persistent problems of forensic psychiatric patients, after positive psychotic symptoms and drug withdrawal have been treated in the acute phase, consist of symptoms that to a certain extent may be likened to the mental fatigue that is seen after a traumatic brain injury, severe infections or cerebrovascular insults (20). Fatigue, lack of initiative, trouble concentrating, stress sensitivity, irritability and a great need for sleep may be understood from many different explanation models, for example, as negative and cognitive symptoms in cases of schizophrenia, reactions after prolonged substance use, institutionalisation, depressive reactions, or as effects of extensive pharmacological treatment. Future research may show if an explanation model based on mental fatigue after some form of condition influencing brain health could give an even more complete picture of the challenges of forensic psychiatric rehabilitation, and if biomarkers for traumatic brain injury (21) could be used for evaluation of the brain health of forensic psychiatric patients.

## Physical Health

The brain is affected in various ways by physical illnesses that can often be investigated and treated. The risk of developing diabetes (22), metabolic syndrome (23) and cardiovascular disease (24) is heightened with psychotic disorders, and patients with a psychotic disorder have an estimated reduced life expectancy of 10–20 years (23–25). Diabetes and metabolic syndrome, which predispose to cardiovascular disease, stroke and premature death, can be detected with simple measurements and blood tests that should be performed for all forensic psychiatric patients. Cardiovascular disease, diabetes and metabolic syndrome are all linked to poorer cognitive functions (26–28). Structured interventions for lifestyle changes, with targets such as the patient giving up smoking or losing weight, can have beneficial effects on physical and mental health (29).

## Cognitive Function

Successful forensic psychiatric rehabilitation requires the patient to be able to use their cognitive abilities. Cognitive defects are a core symptom of schizophrenia that affect the patient’s ability to make decisions and to manage independent living (30). Recently it was shown that attention problems in a group of forensic psychiatric patients correlated with future risk of violence and less rehabilitative engagement (31). Although there is currently no guaranteed effective treatment for cognitive symptoms, experimental treatment with transcranial magnetic stimulation has proven to be able to improve working memory in schizophrenia patients (32) and it has been suggested that pharmacotherapy for substance use could improve executive functions in misuse patients (33). Most interestingly, a randomized controlled trial of cognitive remediation training, a behaviorally based treatment for cognitive deficits in schizophrenia, showed promising effects on cognitive function in a cohort of forensic psychiatric patients with psychotic disorders (34).

## Diet

Many forensic psychiatric patients have unhealthy dietary habits, with too high a calorie intake and an unbalanced diet. It is well known that alcohol misuse affects dietary intake and produces vitamin disturbances, but vitamin disturbances are also seen in psychotic disorder cases and vitamin B supplements could be a future supplementary treatment for schizophrenia in order to reduce symptoms (35). Vitamin D has also been suggested as supplement for psychosis treatment (36) and vitamin D deficiency has been demonstrated in forensic psychiatric patients (37).

## Physical Exercise

Low oxygen uptake ability is an independent risk factor for cardiovascular disease and premature death (38). Low oxygen uptake ability has been demonstrated in patients with a psychotic disorder (39) and in patients in forensic psychiatric care (40). Aerobic exercise offers one possibility for improving patients’ general health and their cognitive functions (8) possibly *via* activation of neurotrophic factors, such as BDNF (brain-derived neurotrophic factor), and brain repair (41) although the mechanism is not fully understood (42). In patients with schizophrenia, aerobic exercise has positive effects on psychotic symptoms, cognitive function, general functional outcomes and quality of life (43, 44). Aerobic exercise can also be expected to reduce the incidence of metabolic syndrome in forensic psychiatric patients and thereby reduce cardiovascular morbidity, diabetes and premature death (45).

## Cognitive Training

Cognitive training and other strategies for facilitating cognition (cognitive remediation) can improve cognitive function in cases of several different mental illnesses such as schizophrenia and substance use syndrome (46) and even in forensic psychiatric patients (34). Patients with schizophrenia who also have metabolic syndrome do not, however, get the same effect from cognitive training as schizophrenia patients without metabolic syndrome (47). Thus, to get an effect from cognitive training, psychosis patients should first be treated for their metabolic syndrome. A particularly interesting thought is therefore to combine various non-pharmacological interventions to achieve the best effect on brain health. In one pilot study, a combined treatment of aerobic exercise and cognitive training produced improved cognitive function in patients with schizophrenia (48).

## Pharmacotherapy

Pharmacotherapy is a staple of forensic psychiatric treatment, and most patients are given antipsychotics (2). Compliance with antipsychotic drug treatment is regarded on the one hand as perhaps the most important factor for avoiding recidivism into serious crime (49) along with freedom from misuse. On the other hand, antipsychotics have side effects that can have an adverse effect on cognition and physical health (50). Although at lower doses antipsychotics are seen as being able to improve cognition in certain patients, the opposite effect can been seen at higher doses, particularly when there is a long treatment period, or when the patient is also taking anticholinergics to deal with extrapyramidal side effects. A structured and cautious reduction of the dose of antipsychotics in forensic psychiatric patients, in a stable clinical condition, can bring about improved cognition and functional outcomes (51). Atypical antipsychotics have been associated with metabolic side effects and the incidence of diabetes (52) and metabolic syndrome, but this is probably true of all types of antipsychotics to a certain extent (50). In one group of schizophrenia patients being treated with olanzapine, the incidence of metabolic syndrome was linked to lower blood levels of the neurotrophic factor BDNF (53). In addition, the future study of clozapine-treatment, a common antipsychotic in forensic psychiatry probably due to its effects on aggressive behavior and treatment-resistant schizophrenia (54), may be of great interest with regard to putatively positive effects on brain health.

## Conclusions and Recommendations

There is much to indicate that forensic psychiatric patients have various complex conditions, which vary from patient to patient, that may potentially affect brain health. These conditions may affect the whole brain either in a general way (relating to, for example, physical health, previous diffuse brain trauma or prolonged substance use) or in a specific way (relating to, for example, psychotic disorders, pharmacotherapy or localised brain injuries), although the dividing line between the two groups is vague. Here we put forward the hypothesis that interventions that improve brain health in forensic psychiatric patients should be able to result in lower degrees of psychiatric morbidity, improved cognitive functions, better physical health, shorter hospital stays, increased independence and a longer life. This approach and these interventions demand a broad range of expertise within the forensic psychiatric team, which in addition to psychiatric and psychological expertise also needs expertise in general medicine, physical exercise and diet management.

From a medical-psychiatric perspective, the patient should be investigated and treated for physical illnesses with particular focus on metabolic syndrome and other risk factors for cardiovascular disease and diabetes. Physical exercise here occupies a special position as a promising form of treatment. Pharmacotherapy should be planned so as to minimise it leading to metabolic and cognitive side effects. Particular focus should be placed on investigating and mapping any brain injuries and their effects. From a psychological perspective, the patient should undergo neuropsychological investigation with particular focus on cognitive functions. These functions should be followed over time and the patient offered cognitive training or targeted support measures. From a care perspective, the patient should be offered as much support as possible in making lifestyle changes concerning diet, exercise and stopping smoking. From a social perspective, forensic psychiatric patients heading towards outpatient care and at the end of care should be offered support, activities and housing suited to their functional level. From a risk assessment perspective, it may be added that one of the hardest challenges for forensic psychiatry is assessing the risk of recidivism into serious crime and communicating that to the courts. Brain-related methods may become part of this risk assessment and risk communication (55–57) which means that forensic psychiatric methods for care, treatment and rehabilitation with a focus on brain health may also be significant for risk assessments and communication with the courts. Finally, we wish to encourage research concerning the mental and physical health of forensic psychiatric patients, with particular focus on conditions affecting brain health. Knowledge concerning longitudinal progress, brain health-related biomarkers and interventions to support brain health would be of particular importance.

## Author Contributions

PA and HB contributed conception and design of the study. PA wrote the first draft of the manuscript. PA and HB contributed to manuscript revision, read and approved the submitted version.

## Funding

This work was supported by the Swedish Research Council for Health, Working Life and Welfare under Grant no. 2018-01409.

## Conflict of Interest

The authors declare that the research was conducted in the absence of any commercial or financial relationships that could be construed as a potential conflict of interest.
